# Recent Advances in Natural-Polymer-Based Hydrogels for Body Movement and Biomedical Monitoring

**DOI:** 10.3390/bios14090415

**Published:** 2024-08-27

**Authors:** Jing Liu, Saisai Li, Shuoze Li, Jinyue Tian, Hang Li, Zhifeng Pan, Lijun Lu, Yanchao Mao

**Affiliations:** Key Laboratory of Materials Physics of Ministry of Education, School of Physics, Zhengzhou University, Zhengzhou 450001, China

**Keywords:** natural polymers, hydrogel, body movement detection, biomedical monitoring

## Abstract

In recent years, the interest in medical monitoring for human health has been rapidly increasing due to widespread concern. Hydrogels are widely used in medical monitoring and other fields due to their excellent mechanical properties, electrical conductivity and adhesion. However, some of the non-degradable materials in hydrogels may cause some environmental damage and resource waste. Therefore, organic renewable natural polymers with excellent properties of biocompatibility, biodegradability, low cost and non-toxicity are expected to serve as an alternative to those non-degradable materials, and also provide a broad application prospect for the development of natural-polymer-based hydrogels as flexible electronic devices. This paper reviews the progress of research on many different types of natural-polymer-based hydrogels such as proteins and polysaccharides. The applications of natural-polymer-based hydrogels in body movement detection and biomedical monitoring are then discussed. Finally, the present challenges and future prospects of natural polymer-based hydrogels are summarized.

## 1. Introduction

With the rapid development of artificial intelligence and people’s rising awareness of the importance health management, a variety of soft and portable flexible electronic products connecting living biological tissues and synthetic electronic systems are emerging [1,2,3,4,5]. Conventional rigid electronic devices suffer from non-stretchable and low-conformability restrictions, which greatly limits their practical applications [6,7]. In contrast, flexible electronic devices have the unique advantages of being portable, lightweight, stretchable and adaptable to many different situations [8,9,10,11,12,13]. Moreover, flexible electronics can be used as a multifunctional interface between medical monitoring devices and the soft body tissues or skin, which has proven to be extremely useful for body movement detection [14,15] and various biomedical monitoring applications such as medical sensors [16] and wearable devices [17,18,19]. Nowadays, many synthetic polymers such as rubber, PI, PET, etc. have been applied to the fabrication of flexible electronics, but these synthetic polymers have the limitation of high Young’s modulus and lack of biocompatibility [20,21,22]. Hence, hydrogels have attracted increasing attention as ideal candidates for flexible electronic devices due to their excellent mechanical properties and intrinsic biological-tissue-like characteristics [23,24].

Hydrogels with three-dimensional mesh structure are usually hydrophilic and soft. With the deepening of research, some hydrogels featuring high stretchability, self-healing [25], excellent adhesion [26] and other advantages are being developed [27,28]. The high mechanical compatibility between hydrogels and biological tissues has attracted research interest in the use of hydrogels for the collection of human physiological signals, such as body movement detection and biomedical monitoring [29,30,31,32,33]. However, conventional synthetic polymer hydrogels have been known to display poor biocompatibility, difficult degradation and weak skin adaptability. Flexible electronic devices based on these hydrogels usually exhibit problems such as low impedance and interface mismatch between the skin and the device [34,35,36,37,38]. Their non-biodegradability and low biocompatibility may be harmful to human health. Therefore, it is imperative to develop sustainable, economical and environmentally friendly natural hydrogels [39,40,41,42,43,44].

Common natural polymers, such as proteins, polysaccharides and natural rubber, are very important renewable resources [45,46,47,48]. Generally, natural polymers are produced in different kinds of organisms like microorganisms, animals and plants. Compared to most synthetic polymers, they have advantages of low cost, non-toxicity, biodegradability and eco-friendliness [49,50,51,52]. As environmental issues, safety concerns and sustainable development receive growing attention, there is a strong trend to produce hydrogels from environmentally friendly and biocompatible natural polymer materials [53,54,55,56]. The development of sustainable and biodegradable natural-polymer-based hydrogels can markedly alleviate the environmental problems and health risks from waste materials [57,58,59,60]. At present, many reports have proposed hydrogels based on natural polymers as basic materials. In addition, natural-polymer-based hydrogels are a very promising material that has been proven to have very significant applications in the use of flexible electronics for detecting body movement and biomedical monitoring [61,62,63,64].

In this review, we aim to provide a complete overview of natural-polymer-based hydrogels for human motion detection and biomedical monitoring. We begin with a general introduction to natural-polymer-based hydrogels to give the reader a basic understanding of natural polymers. We then review the applications in flexible electronics and other applications of natural polymer hydrogels, including proteins such as gelatin, filipin proteins, etc., and polysaccharides such as cellulose, agarose and hyaluronic acid. Subsequently, we highlight the latest research progress and status of natural-polymer-based hydrogels for human movement detection and biomedical monitoring, including electrocardiogram (ECG), electromyogram (EMG) and electroencephalogram (EEG), in recent years. Finally, the present challenges and new directions in the development of natural-polymer-based hydrogels are summarized and discussed. This review is intended to help provide a more comprehensive understanding of this field and advance it to the next level.

## 2. Natural-Polymer-Based Hydrogels

Natural polymers are high-molecular-weight compounds existing in natural materials or tissues. Hydrogel networks from these natural polymers have high safety and unique properties such as renewability, non-toxicity, high biocompatibility, biodegradability and high water-holding capacity. Therefore, natural-polymer-based hydrogels have received growing attention in recent years [65,66,67,68]. Currently, many natural polymers, including proteins and polysaccharides [69], have been used to prepare hydrogels with strengthened mechanical and electrical properties, which have great potential as biomaterials [70]. However, other natural polymers have been studied relatively little with respect to hydrogels. Therefore, this section focuses on recent research advances in natural protein- and polysaccharide-based hydrogels.

### 2.1. Protein-Based Hydrogel

**Figure 1:**
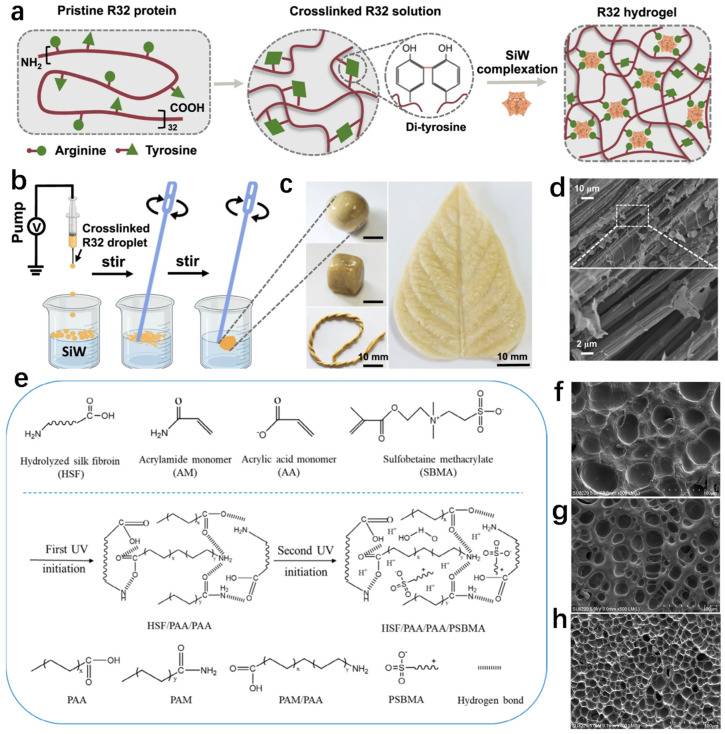
The process and characterization of the preparation of various protein-based hydrogels. (**a**) Proposed design principle of the R32 hydrogels. (**b**) Schematic illustration of the process for complexation between crosslinked R32 and SiW. (**c**) The as-prepared ball-like hydrogel can be remolded into different shapes, such as a cube, a rope or a leaf. (**d**) SEM images of the microfibril structures in the hydrogels [75]. (**e**) Reaction mechanisms of HSF/PAM/PAA/PSBMA. (**f**) SEM images of PAM/PAA hydrogel. (**g**) SEM images of HSF8%/PAM/PAA hydrogel. (**h**) SEM images of HSF8%/PAM/PAA/PSBMA2% hydrogel [81].

Peptide- and protein-based hydrogels are a promising group of biomaterials [71]. Proteins exhibit functional characteristics and remarkable structures suitable for the manufacture of hydrogels, such as biocompatibility and biodegradability. Proteins contain amine and carboxylic acid functional groups, which can be aggregated by proteins to form a gel network with water-holding capacity and stabilized by non-covalent or chemical cross-linking. Therefore, the protein-based natural polymer hydrogel with higher mechanical stability and controllable degradation rate under physiological environment can be obtained [72,73]. Natural conductive proteins are potential material candidates for use as artificial skin [74]. Based on the great advantages of protein-based hydrogels, Xia et al. have developed a magnetically responsive and photothermal soft robot that can perform a range of complex tasks such as repairing artificial blood vessels [75]. The R32 protein consists of 32 repeating resin-like blocks. A hydrogel containing many charged amino acids and tyrosines was formed by complexing the R32 protein with silicotungstic acid (SiW), as shown in Figure 1a. The complexation process includes two steps as shown in Figure 1b; the first step is dropping the pre-crosslinked R32 protein solution into the SiW solution, and the second step is gentle stirring, which ultimately obtains a protein hydrogel showing shape plasticity at room temperature, as shown in Figure 1c. This property facilitates the seamless contact of this hydrogel with other substrates. The scanning electron microscope (SEM) image of the microfiber structure of the hydrogel is shown in Figure 1d, indicating that this hydrogel has a microfibrillar structure similar to many natural underwater adhesives. The resulting adherent hydrogel has a very high potential in biomedicine due to its rapidly switching between a non-adherent rigid state and an adherent soft state within a narrow and mild temperature range.

In addition, natural silk protein extracted from the cocoons of domestic silkworms is another of the most promising proteins, being strong, sustainable, natural, low-cost, biodegradable and biocompatible [76,77,78,79,80]. Based on the characteristics of silk proteins, Xu et al. proposed a method to prepare a dual-network-structure silk-protein-based tough hydrogel with antimicrobial activity and ionic conductivity (HSF8%/PAM/PAA/PSBMA2% hydrogels) by a two-step UV polymerization [81]. The hydrogel has high mechanical strength and electrical conductivity and can be used to monitor many tiny deformation movements. The reaction mechanism of this silk fibroin (SF)-based strong hydrogel during the preparation process involving the dual network structure is shown in Figure 1e. In addition, the SEM images shown in Figure 1f–h indicate that the HSF8%/PAM/PAA/PSBMA2% hydrogel has a dense and uniform three-dimensional network with smaller pore size compared to the other two hydrogels mentioned. This study provides a relatively simple method to prepare a silk-protein-based conductive hydrogel with high strength and multifunctionality, and the hydrogel can also be used as a flexible strain sensor to monitor body joint activities. Furthermore, Chen et al. proposed a simple method to prepare strong silk protein hydrogels by adding surfactants to silk protein solution. The prepared silk protein hydrogels had excellent mechanical properties while maintaining good biocompatibility [78]. Liu et al. controlled the degumming time of the silk proteins obtained from raw silk textile yarns to prepare a silk protein hydrogel network [80]. In conclusion, silk-protein-based strong hydrogels have very broad application prospects in flexible electronics.

**Figure 2:**
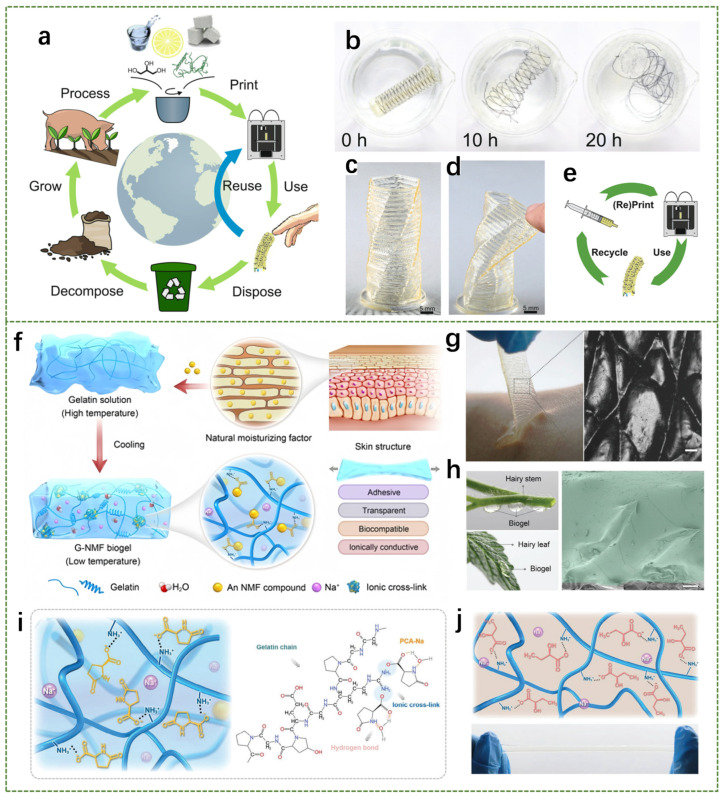
Preparation process and characterization of a variety of gelatin-based hydrogels. (**a**) The bio-ecological recycling routes. (**b**) When immersed in water, the gelatin actuator and waveguides swell and dissolve. (**c**) Printed twisted vase with a wall thickness of 0.8 mm. (**d**) The soft structure and low Young’s modulus allow reversible deformation. (**e**) Printed parts can be recycled by heating and reprinting the material [86]. (**f**) Schematic illustration of the biogel fabrication process. (**g**) Photograph and optical image of the biogel after in situ gelation. (**h**) Photographs and cross-sectional SEM image of the biogel applied on hairy plants. (**i**) Schematic illustration of the interaction between gelatin chain and PCA-Na. (**j**) Schematic illustration of the interaction between gelatin chain and sodium lactate [87].

Besides the two proteins mentioned above, gelatin is a biodegradable protein obtained by acid and alkali processes on collagen [81]. The combination of biodegradable gelatin with hydrogels to form gelatin-based composite gels is receiving more and more attention due to its wide range of applications [82,83]. The materials involved in soft robotics often come from non-renewable or non-biodegradable resources, and soft materials have a limited lifespan or cannot be recycled and reused [84,85]. Therefore, the development of sustainable soft actuators and sensors that are renewable, biodegradable and resource-recyclable is crucial. On this basis, Hartmann et al. reported a customized 3D printing process that designs gelatin-based hydrogel inks into dimensionally stable, complex objects [86]. This process achieves zero waste and recyclability for electronic skin applications. Besides printing biodegradable gels, the sustainability of the technology is maintained by establishing bio-ecological recycling routes, shown in Figure 2a. Since the printed gel is biodegradable and can be directly dissolved in water (Figure 2b), the printed biogel can be reused easily and does not cause environmental pollution. The properties of low modulus and soft structure enable the gel to be reversibly deformed (Figure 2c,d). In addition, the printed biogel also has thermal reversibility, as shown in Figure 2e, which allows an obsolete or damaged biogel device to be cycled through by reheating the material and printing a new one. Apart from this, Ying et al. proposed a general strategy for the preparation of functional hydrogels based on gelatin and sodium pyrrolidone carboxylate [87]. The biogels produced by this strategy are highly flexible, resistant to dehydration and ionic conductivity and fully degradable. Figure 2f depicts the preparation process of this biogel, in which the PCA-Na was directly doped into the gelatin solution, and then the mixture was cooled down to room temperature to finally obtain a biogel with ionic conductivity, high mechanical strength, and long-term anti-dehydration properties. It was demonstrated that when the biogel was applied to the skin, it conformed to the microscopic curvature of the skin texture before gelation, as shown in Figure 2g. In addition to applications in human skin, the gel has great potential in establishing a robust interface between plants and electrodes (Figure 2h). The principle of intermolecular interaction between gelatin and PCA-Na is shown in Figure 2i, and the principle of gelatin-sodium-lactate interaction is shown in Figure 2j. The result shows that NMF compounds, such as sodium lactate and PCA-Na, which contain mobile ions and carboxylate groups, are the optimal additives for the preparation of the all-natural gelatin-based biogel. In summary, gelatin-based hydrogels have very wide application prospects in facilitating long-term high-fidelity electrophysiological signal recording and constructing reliable biological interfaces.

### 2.2. Polysaccharide-Based Hydrogel

Polysaccharides are a group of abundant renewable natural materials with superior biocompatibility and biodegradable properties and excellent biological properties, which are highly suitable for the preparation of biomimetic hydrogels [88,89,90]. The rich functional groups and unique structure of polysaccharide-based hydrogels give them unique physicochemical properties and excellent mechanical properties [91]. Polysaccharide-based conductive hydrogels have attracted wide interest as smart biomaterials in wearable devices.

**Figure 3:**
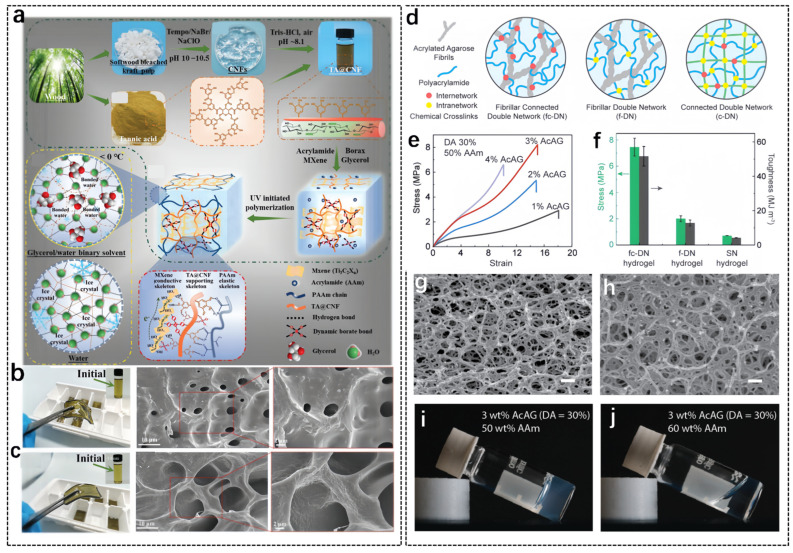
Preparation process and characterization of cellulose-based hydrogels and agarose-based hydrogels. (**a**) Schematics illustrating the preparation of the TA@CNF and schematics illustrating the formation of TA@CNF/PAAm/MXene interpenetrating networks in the organohydrogel. (**b**,**c**) Photograph of the PTCM-Gly5 and PTCM-Gly1 organohydrogel and the inset of initial raw material mixtures. SEM images of the freeze-dried PTCM-Gly5 and PTCM-Gly1 organohydrogel [94]. (**d**) Schematic illustrations for three different DN topologies in hydrogels. (**e**) Tensile stress–strain curves of AcAG/PAAm fc-DN hydrogel, AG/PAAm f-DN hydrogel and PAAm SN hydrogel. (**f**) A comparison of fracture strength and toughness of the three types of hydrogels. (**g**) SEM images of 3 wt% AG gels after freeze-drying. (**h**) SEM images of 3 wt% AcAG gels after freeze drying. (**i**) Photos of AcAG/AAm solutions containing 3 wt% AcAG (DA = 30%) and 50 wt% Aam. (**j**) Photos of AcAG/AAm solutions containing 3 wt% AcAG (DA = 30%) and 60 wt% AAm [99].

Cellulose, as an organic and renewable resource, is a long-chain polymer polysaccharide formed by a series of glucose units which widely exists in many plants and has excellent characteristics including low price, non-toxicity, renewability, etc. [92]. Moreover, cellulose is an ideal raw material for hydrogels as it contains a large number of hydrophilic hydroxyl groups [93]. Therefore, the development of cellulose-based hydrogels has attracted an increasing amount of research interest. Recently, Kuang et al. developed conductive nanocomposite organohydrogel-based cellulose nanofibrils (designated as PTCM-Gly) [94], which showed very excellent freezing resistance, stretchability, high sensitivity, reversible adhesion and long-term environmental stability. During the preparation process of PTCM-Gly, shown in Figure 3a, cellulose nanofibrils (CNFs) were firstly extracted from sulphated pulp, and then tannic-acid-decorated cellulose nanofibrils (TA@CNF) were obtained by modifying tannic acid (TA) primer on the surface of the CNFs. Next, conductive MXene nanosheets and glycerol were added into the polymer bi-network composed of polyacrylamide (PAAm) and TA@CNF to finally obtain PTCM-Gly organic hydrogel. This hydrogel has a uniform color and high light transmittance (Figure 3b,c). The final PTCM-Gly organic hydrogel can be assembled into flexible wearable strain sensors for large and tiny movement detection. In addition, Hinestroza et al. obtained a tough cellulose hydrogel by means of stepwise solution exchange. The cellulose hydrogel was able to maintain its original shape despite changes in temperature and pH, with tough, long-lasting biodegradable characteristics [93]. In summary, cellulose is widely used in the preparation of hydrogels due to its outstanding biological properties.

Agarose [95] is another typical polysaccharide, consisting of a linear arrangement of polysaccharide galactans [96]. Upon heating and cooling, it can form a unique network of double helical structures [97]. Furthermore, in some polysaccharide hydrogels, mixing with agarose can also remarkably enhance their mechanical properties [98]. Zhang et al. proposed a strategy to strengthen hydrogels using fiber-connected dual networks (fc-DN) [99]. The obtained fc-DN hydrogels showed a significant improvement in both toughness and strength compared to conventional hydrogels, and still maintained low plastic deformation. The fc-DN hydrogels differ from conventional fibrillar dual network (f-DN) hydrogels or connected dual network (c-DN) hydrogels by a fibrous first network and an elastic second polymer network chemically linked through covalent bonds (Figure 3d). The experimental results showed that the mechanical properties of fc-DN hydrogels were significantly improved (Figure 3e,f). SEM images of 3 wt% AG (agarose) gel and 3wt % AcAG (acrylated agarose) gel are shown in Figure 3g,h, where it can be seen that acrylation does not seem to affect the agarose raw fiber microstructure. Figure 3i,j indicate that the high concentration of acrylamide (AAm) (60 wt%) hindered the gelation of AcAG. In summary, the design of fc-DN provides a new toughening mechanism with a wide range of applications in electronic skin. Wei et al. obtained a self-healing conductive hydrogel by heating, mixing and then cooling agarose in combination with Li^+^. The resulting hydrogel with good mechanical properties, self-healing and electrical conductivity was used in strain sensors for movement detection of finger sliding [97]. Koo et al. presented a transparent conductive hydrogel synthesized based on agarose polymers with high water retention and good self-healing properties, which was used to fabricate a very flexible pressure sensor [96]. In conclusion, the addition of agarose in the preparation of hydrogels can effectively improve the various biological properties of hydrogels, and natural-polysaccharide-based hydrogels have a very bright application prospect.

**Figure 4:**
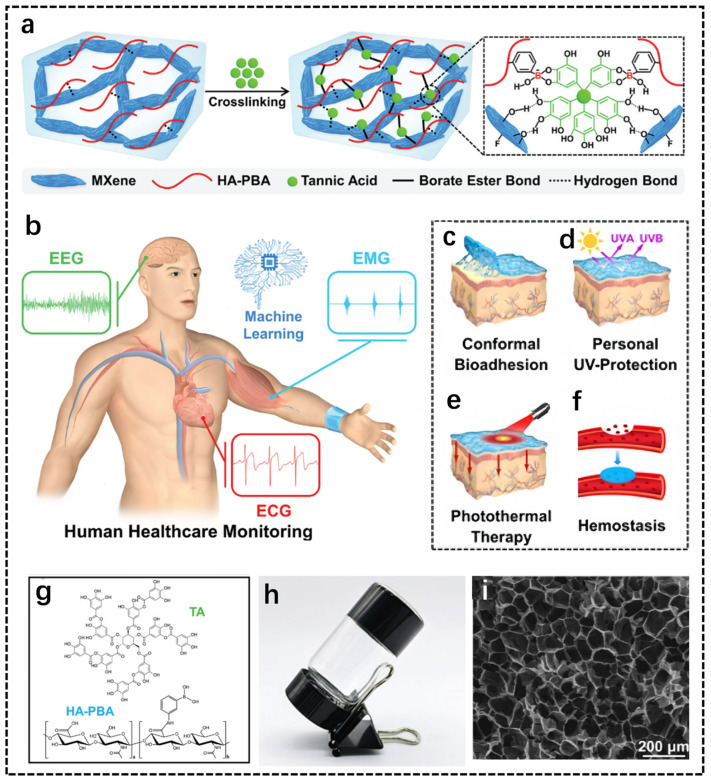
Preparation process and characterization of hyaluronic acid polysaccharides. (**a**) Schematic illustration of the preparation of the MXene/HA-PBA/TA hydrogel. (**b**) The further promising applications in human healthcare detection. (**c**) Conformal bio-adhesion. (**d**) Personal UV protection. (**e**) Photothermal therapy. (**f**) Wound hemostasis. (**g**) Molecular structure of TA and HA-PBA. (**h**) The optical image of the MXene/HA-PBA/TA hydrogel. (**i**) SEM image of the freeze-dried MXene/HA-PBA/TA hydrogel [101].

In addition to the two polysaccharides mentioned above, hyaluronic acid (HA) as one of the common polysaccharides is a linear polysaccharide and has been widely used in the design of hydrogels owing to its large number of reactive group modification sites and biofunctionality [100]. Recently, Zhang et al. proposed a method for the preparation of the HA-based hydrogel via the assembly of MXene (Ti_3_C_2_Tx) nanosheets [101]. The obtained hydrogels exhibited excellent sensing properties, remarkable adhesion and high electrical conductivity. In the preparation method shown in Figure 4a, MXene nanosheets were introduced into the polymer network containing phenylboronic-acid-grafted hyaluronic acid (HA-PBA) and tannic acid (TA), and finally the multifunctional bioadhesive MXene/HA-PBA/TA hydrogel was obtained. The multifunctional sensors assembled from MXene/HA-PBA/TA hydrogels can be used for human health monitoring, conformal bio-adhesion, personal UV protection, photothermal therapy and wound hemostasis (Figure 4b–f). The molecular structural formulae of TA and HA-PBA are also shown in Figure 4g. Optical images and SEM images of the MXene/HA-PBA/TA hydrogel are shown in Figure 4h,i, indicating that the hydrogel has a three-dimensional porous microstructure, allowing it to aid the transport of conductive ions and electrons in the conductive pathways inside the hydrogel. The hydrogel can also be stably affixed to human skin for high-quality recording of various electrophysiological signals. Serafin et al. synthesized a conductive hydrogel by combining polypyrrole nanoparticles with HA hydrogel [102]. This conductive hydrogel has very excellent mechanical properties coinciding with the mechanical properties of the spinal cord and has also been used for electroactive tissue engineering due to its good electrical conductivity and excellent biocompatibility. In summary, as one of the common polysaccharides, HA can be combined with various other polymers to produce hydrogels showing excellent properties for many applications, attracting much attention to these HA-based hydrogels.

## 3. Applications

Protein- and polysaccharide-based hydrogels with excellent biocompatibility, good electrical conductivity and other properties show great potential for application in flexible electronics and other fields. The outstanding flexibility and degradability allow them to be used for the preparation of flexible sensors that can be attached to the skin surface for real-time movement monitoring of human body data [103]. Meanwhile, natural-polymer-based hydrogels can be fabricated into epidermal electrodes for the collection of electrical signals generated by muscle activity, cerebral cortex activity, heart activity, etc., to monitor human physiological electrical signals in real time. The wide range of applications in human movement detection and biomedical monitoring has attracted considerable interest [104].

### 3.1. Body Movement Detection

**Figure 5:**
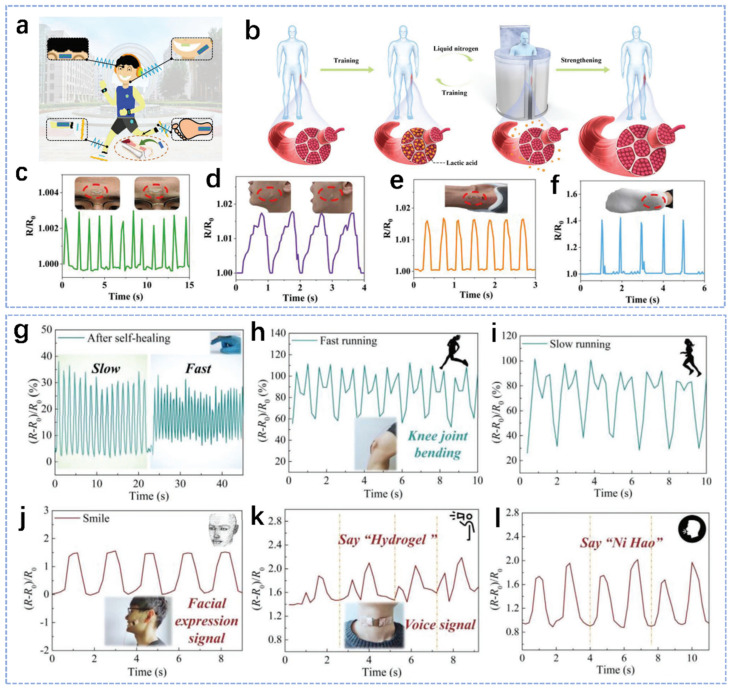
Description and schematic of the application of various natural-polymer-based hydrogels for use in body movement detection. (**a**) Schematic illustration of strain sensing tests on different body parts. (**b**) Schematic diagram of human muscle strengthening. Real-time resistance changes when adhered to the middle of (**c**) the forehead, (**d**) cheek, (**e**) heel, and (**f**) sole [105]. (**g**) Finger-bending signal detection of the organohydrogel-based sensor. (**h**,**i**) Demonstration of the organohydrogel-based sensor in response to knee movement detection. Demonstration of the organohydrogel-based sensor for (**j**) facial expression and (**k**,**l**) voice detection [94].

With the increasing demand for and interest in wearable flexible electronic devices in recent years, flexible hydrogel thin-film sensors with high sensitivity and strength have great application prospects in human motion detection [105,106,107]. Among them, natural-polymer-based hydrogel film sensors have adjustable mechanical properties, extensive bonding capabilities, high biocompatibility and excellent reactivity to functional modifications [108]. They are ideal materials for the development of flexible wearable sensors, which can be used for real-time detection of various large or small human movements [109,110,111,112,113]. Therefore, natural-polymer-based hydrogel sensors are very promising as electronic skins for motion detection [114,115,116]. Recently, Yao et al. proposed a strategy inspired by human muscle training for the preparation of a gelatin-based tough hydrogel (GBTH) [105]. The obtained GBTHs showed significant toughness, biocompatibility, in vivo self-degradability and similarity to natural tissue components. During muscle training, the human body produces a large amount of lactic acid, the excessive accumulation of which has a negative effect on the human body. Therefore, professional athletes may use liquid nitrogen for rapid cooling to quickly release the excessive lactic acid produced by exercise to strengthen their muscle fibers, as shown in Figure 5a. For the preparation process of hydrogel, firstly, the hydrogel is placed in salt solution and mechanically trained to produce ${SO}_{4}^{2-}$, which reorders the interior of the hydrogel and increase its crystallinity. Then the trained hydrogel is immersed in phosphate-buffered saline (PBS). At that time, ${SO}_{4}^{2-}$ is discharged from the hydrogel again. This process is analogous to the liquid nitrogen treatment performed after exercise as described above. After several iterations of these processes, the bonding between the chains inside the hydrogel becomes stronger and stronger, resulting in the formation of a tough hydrogel. Furthermore, liquid metal injected into the tough GBTH can be used to monitor various parts of the human body (Figure 5b). Because partial deformation leads to changes in electrical resistance, the liquid-metal-based tough hydrogel can sense the change in electrical resistance and generate feedback accordingly, and thus it can be used for all-around real-time motion detection of human health, as shown in Figure 5c–f. This strategy provides a new way to prepare highly biocompatible hydrogels for real-time detection of body motion. In addition, Kuang et al. have elaborated a nanocomposite organic hydrogel, PTCM-Gly hydrogel [94]. The sensor of PTCM-Gly organic hydrogel can detect the movement behavior of each joint, and can also accurately monitor the complex movements of the knee joint, as in Figure 5g–i. In addition, some tiny human movements like smiling and speaking can be precisely detected, as shown in Figure 5j–l.

**Figure 6:**
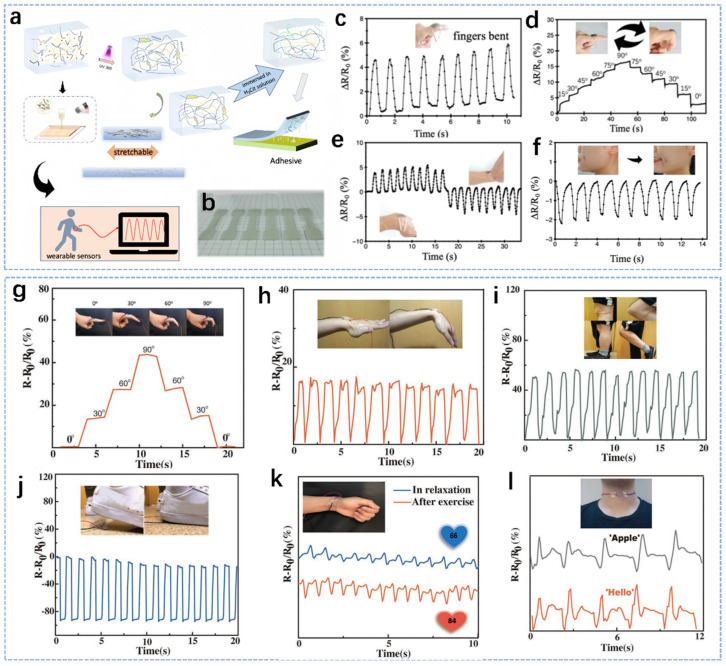
Description and schematic of the application of various natural-polymer-based hydrogels for use in body movement detection. (**a**) Preparation of acrylamide/chitosan composite hydrogel. (**b**) 3D-printed dumbbell model. (**c**) Human motion detection of fingers bending. (**d**) Human motion detection of continuous finger movement at different angles. (**e**) Motion detection of the wrist. (**f**) Detection of smile [121]. (**g**–**i**) The relative resistance changes of sensors versus time for real-time monitoring of various human motions. (**g**) Bending and releasing of forefinger, (**h**) wrist and (**i**) knee. (**j**) Standing and walking position of foot. (**k**) Sensing the subtle muscle movements of the radial pulse of wrist at ease and after exercise. (**l**) Sensing of subtle muscle movements of the throat as the same person says the words “Apple” and “Hello” five times [122].

Flexible wearable sensors based on natural polymer hydrogels have received increasing attention due to their great potential in the fields of e-skin, medical monitoring and soft robotics [117,118,119,120]. Maimaitiyiming et al. prepared a hydrogel ink based on polyacrylamide and chitosan [121]. The hydrogel ink has high mechanical properties, electrical conductivity and good self-adhesion, and it can also be used to prepare flexible sensors for human motion detection by 3D printing technology (Figure 6a). Such 3D-printable hydrogel inks enable the preparation of hydrogel models with different structures (Figure 6b). Based on the combined advantages of electrical conductivity, high stretchability, self-adhesion, 3D printability, sensing stability and high sensitivity, hydrogels can adhere to every moving part of the body for detection of real-time movements in daily life, such as finger movements (Figure 6c,d), wrist movements (Figure 6e), and smiles (Figure 6f). In addition, Jiang et al. proposed a simple method to prepare a highly stretchable, electrically conductive, tough and transparent ion-conducting organohydrogel (PVA–CNF) based on cellulose nanofibers [122]. The PVA–CNF hydrogel has a very high sensitivity to both tensile and compressive deformations, the multifunctional sensors assembled from the PVA–CNF organohydrogel have high sensitivity, durability and stability, and they can be used to detect human body movement. The strain sensor based on PVA–CNF organic hydrogel, integrated into different joints of the human body, can detect complex body movements in real time. Due to the high sensitivity of the strain sensors, it is possible to detect the bending behavior at different bending angles in various parts of the body, such as fingers (Figure 6g), wrists (Figure 6h) and knees (Figure 6i). Furthermore, pressure sensors based on PVA–CNF organohydrogels can also be used to detect walking motion through the integrated sole (Figure 6j). The pressure sensors can also respond in a very short time due to their high sensitivity and shape recovery properties. In addition to extensive motion detection, these sensors can also be used to detect subtle motions, such as radial pulses on the wrist (Figure 6k) and throat movement when speaking (Figure 6l). In summary, the PVA–CNF organic hydrogel has a very great application future in the real-time detection of human motion through wearable devices.

Feng et al. produced a dual-network hydrogel based on chitosan, with high tensile properties and excellent self-repairing properties, used as a strain sensor for real-time detection of human movement, including finger movement, throat movement, etc. [123]. Ren et al. prepared a hydrogel based on the natural macromolecular polysaccharide gum arabic with stretchability and excellent self-adhesion, which has great prospects for skin-attachment sensors and has been successfully used for detecting human movement [106]. Xu et al. prepared a starch-based conductive hydrogel which has good mechanical properties and electrical conductivity and fast self-healing ability both underwater and in the air. Based on these advantages, the hydrogel has been used as a highly sensitive strain sensor for sensing very small movements such as swallowing and articulation [111]. Fan et al. have constructed a multifunctional hydrogel based on gelatin by combining chemical cross-linking and physical interactions. The multifunctional hydrogel has high stretchability, good electrical conductivity, self-healing properties and excellent adhesion, and is currently being used as a wearable detector for more comprehensive human health monitoring [110]. In conclusion, natural-polymer-based hydrogels are widely used in human movement detection owing to their excellent properties.

### 3.2. Biomedical Monitoring

Health has always been a concern for everyone. While traditional sensors play a role in health monitoring and other aspects, they have the drawbacks of excessive weight, poor portability and high cost [124,125]. Wearable flexible sensors can solve some of the above problems, but they have their own problems of skin irritation and weak adhesion [126,127]. Considering that the choice of material strongly influences the performance of flexible sensors, several existing natural polymers offer the enormous advantage of being renewable, biodegradable and resource-efficient [128]. Wearable sensors assembled from natural-polymer-based hydrogels have good breathability, adaptability, water retention, antimicrobial activity, biocompatibility and biodegradability [129,130,131,132]. They are also capable of providing stable and repeatable electrical signals, making them invaluable for applications in monitoring electrophysiological signals and health detection, etc. [133,134,135,136]. In this section, the use of natural-polymer-based hydrogels for recording bioelectrical signals, including EMG, ECG and EEG, is reviewed.

#### 3.2.1. EMG

**Figure 7:**
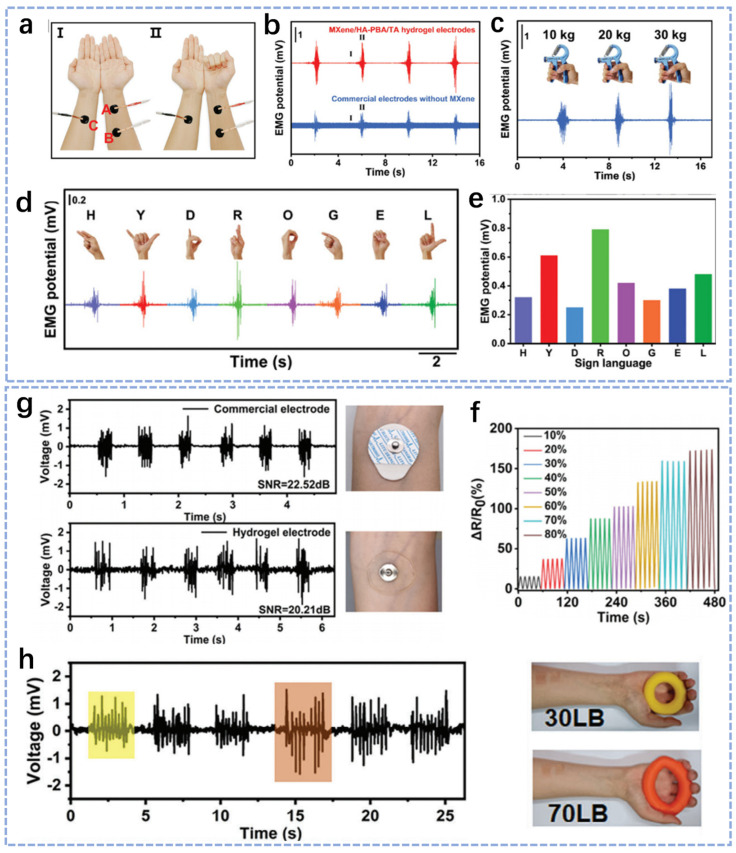
Characterization of the properties of a variety of natural-polymer-based hydrogels with application in EMG monitoring. (**a**) Schematic of the setup for EMG detection: (I) initial, (II) after clenching the fist. (**b**) EMG signals measured by the MXene/HA-PBA/TA hydrogel electrode and commercial hydrogel electrode without MXene ((I) initial, (II) after clenching right fist), respectively. (**c**) EMG signals measured while increasing gripper force. (**d**,**e**) EMG signals collected from the performances of different sign language gestures and those signals’ amplitude. (**f**) Relative resistance change curves of the hydrogel sensor under different tensile strains, ranging from 10% to 80% strain [101]. (**g**) Comparison of EMG signals from commercial electrodes versus hydrogel electrodes. (**h**) EMG signals collected from the hydrogel electrodes when the hand grips and unclenches the two grips with weights of 30 and 70 lb, respectively [140].

The EMG signal usually requires continuous monitoring over a long period and plays an important role in a variety of applications, including muscle health assessment, rehabilitation, etc. A number of signal pollutants such as noise, interference and artifacts as well as loose contact between electrodes and skin can degrade the quality of the EMG signal [137,138]. In recent years, the use of natural-polymer-based hydrogels as epidermal electrodes for real-time acquisition of high-quality EMG signals has attracted increasing attention [139]. As natural-polymer-based hydrogels have strong adhesion and good mechanical properties, they can be directly attached to the skin surface to achieve flexible deformations and human co-conformity [140,141]. Moreover, their low interfacial impedance enables them to accurately monitor weak EMG signals [142,143]. Therefore, natural-polymer-based hydrogels have very strong application prospects to be used as epidermal electrodes for real-time acquisition of EMG signals. Recently, Zhang et al. designed an MXene/HA-PBA/TA hydrogel as the conformal bio-based hydrogel epidermal sensor [101]. This hydrogel sensor with a high signal-to-noise ratio, low modulus, low interfacial impedance and good self-adhesive properties can be stably adhered to human skin and record various electrophysiological signals in high quality, including EMG signals. The researchers detected the changes in EMG signals induced by muscle movements before and after fist clenching by coating MXene/HA-PBA/TA hydrogel electrodes on the arms of volunteers (Figure 7a). The impedance of this hydrogel interface was lower than that of the commercial electrodes (Figure 7b). In addition, the volunteers were asked to hold the grip dynamometer with different forces, and a clear EMG signal could be obtained, as shown in Figure 7c. Moreover, they found that the MXene/HA-PBA/TA hydrogel sensor coated on the volunteer’s arm could monitor the EMG signals of eight different gesture language movements with different amplitudes, as shown in Figure 7d,e, showing great possibilities in helping hearing-impaired people to communicate. Yin et al. prepared a hydrogel by one-pot polymerization using gelatin as raw material [140]. The hydrogel exhibits transparency, stretchability, electrical conductivity, self-adhesion and reusability. Furthermore, it is fast-responsive and strain-sensitive, providing a stable electrical response over a wide range of strain, as shown in Figure 7f. The quality of the EMG signals obtained from this hydrogel for volunteer EMG detection was comparable to the commercial electrodes, as shown in Figure 7g. The researchers found that the EMG signals were correspondingly enhanced with the increase in grip strength, as shown in Figure 7h. As a result, sensors made of natural-polymer-based hydrogels have great future applications in biomedical monitoring. Cui et al. made a novel gelatin-based adhesive hydrogel with excellent adhesion, self-repairing and mechanical properties, which was made into an adhesive electrode for EMG monitoring of finger movements [141]. Qin et al. made a conductive hydrogel by adding silk-protein-modified MXene into polyacrylamide, which makes the hydrogel highly conductive and also ensures good adhesion properties and high tensile properties. Due to its high conformal adhesion properties and low impedance properties, the hydrogel can be made into a bioelectrode for electrophysiological signal monitoring, and the high sensitivity of the hydrogel makes it possible to monitor EMG signals from a wide range of small movements [139]. In conclusion, natural polymers are made into hydrogel bioelectrodes and widely used for EMG signal monitoring owing to their excellent adhesion, conductivity and biocompatibility properties.

#### 3.2.2. ECG

**Figure 8:**
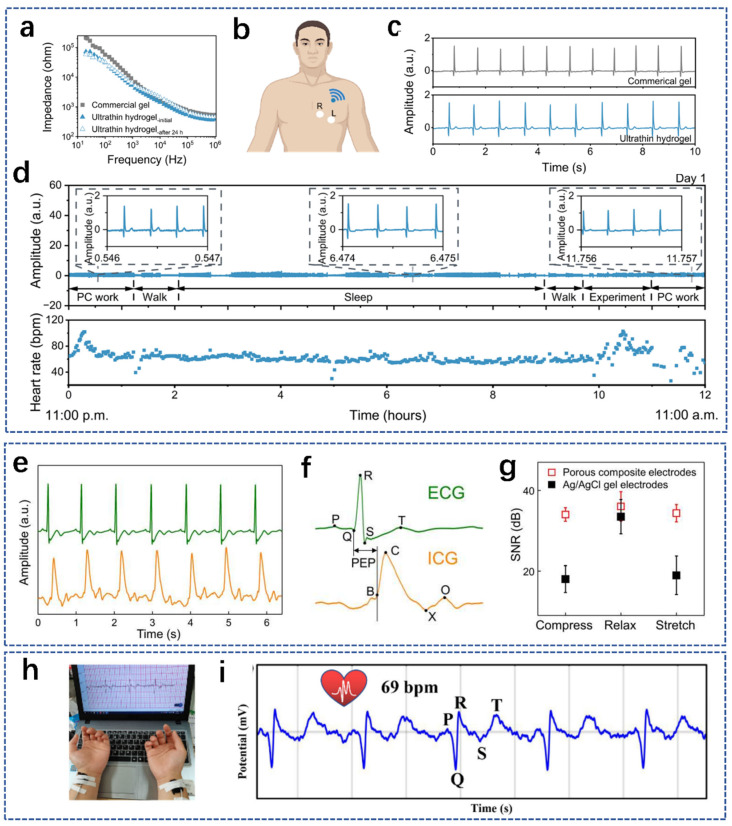
Characterization of the properties of various natural-polymer-based hydrogels and their application in ECG monitoring. (**a**) Skin–electrode contact impedance analysis of commercial gels and ultra-thin hydrogels. (**b**) Schematic illustration of the experimental setup for wireless ECG measurement. (**c**) ECG signals recorded by commercial gels (top) and ultra-thin hydrogels (bottom) in a sedentary state. (**d**) ECG signals monitoring (top) and the heart rate results (bottom) by the ultra-thin hydrogels during various normal daily activities on day 1 from 11:00 a.m. to 11:00 p.m. [145]. (**e**) Concurrent ECG recording using skin-interfaced bioelectronics based on porous composites. (**f**) Magnified ECG signals. (**g**) Signal-to-noise ratio of ECG signals recorded with porous composite electrodes and Ag/AgCl electrodes when stretching, relaxing and compressing the electrodes [153]. (**h**) Photographs of real-time monitoring of ECG curves. (**i**) ECG spectra of the volunteer obtained from the hydrogel bioelectrodes [154].

ECG is a common, convenient and broadly used technique for detecting arrhythmias and other cardiovascular diseases [144,145]. Wearable ECG monitoring is widely prescribed for postoperative recovery and cardiac medication monitoring [146]. The wearable ECG data collection suffers from a variety of noise interferences, including motion artifacts, which are an important source of noise in ECG recordings [147,148]. Motion artifacts can be suppressed by optimizing the contact between skin, clothing and electrodes to reduce the relative motion between the electrodes and the body [149,150]. Therefore, the emergence of natural-polymer-based hydrogel sensors with biocompatibility, degradability, electrical conductivity and good self-adhesion has attracted a lot of attention [149]. These can be used as soft electrodes in ECG devices to minimize the contact impedance between skin and electrodes and achieve wireless real-time heart rate monitoring in any state to obtain high-quality ECG signals [151,152]. Recently, Wang et al. designed an ultra-thin, breathable gelatin-based hydrogel sensor [145]. The hydrogel sensor can self-adhere to the skin and perform high-quality, high-precision electrophysiological monitoring for eight consecutive days. They compared the epidermal electrode impedance of the ultra-thin breathable hydrogel with the commercial gel and found that the epidermal resistance based on the ultra-thin hydrogel was lower than the commercial gel’s (Figure 8a). They also found that the ultra-thin breathable hydrogel had a similar waveform and a lower signal-to-noise ratio than the commercial gel (Figure 8c). In addition, continuous ECG signals of the subjects were monitored while they performed various daily activities (Figure 8d). Figure 8b shows the placement of the ultra-thin breathable hydrogel during ECG monitoring. Yan et al. reported a composite material based on epsilon polylysine [153]. The composite has excellent tensile, breathable, conductive and antimicrobial properties. Real-time, high-fidelity recording of ECG during motion can be performed (Figure 8e,f), and stable, high signal-to-noise-ratio recording of physiological signals under dynamic deformation can also be obtained (Figure 8g). Xu et al. prepared an ionic conductive hydrogel based on theophylline-modified chitosan [154]. The ionic conductive hydrogel has excellent adhesion properties, toughness and fast self-healing, and it can be used as a bioelectrode for ECG signals, as shown in Figure 8h,i. Yu et al. developed a hydrogel by mixing bovine serum protein with other polymers. With good biocompatibility, excellent mechanical properties, self-recovery properties and electrical conductivity, this hydrogel was used as a soft electrode in ECG equipment and showed reliability and stability in heart rate monitoring [144]. Pitsalidis et al. combined gelatin and chitosan with a variety of conductive materials to make a hydrogel which was thermally reversible, conductive, and skin-friendly, and was assembled into hydrogel electrodes for ECG signal monitoring [151]. Gao et al. proposed a method to increase the adhesion of the hydrogel by adding branched starch. The hydrogels with the addition of branched starch had excellent adhesion and were used to make epidermal electrodes with high sensitivity for real-time collection of high-quality ECG signals [137]. To sum up, hydrogels prepared from natural polymers based on proteins and polysaccharides have excellent applications in epidermal electrodes for ECG monitoring.

#### 3.2.3. EEG

**Figure 9:**
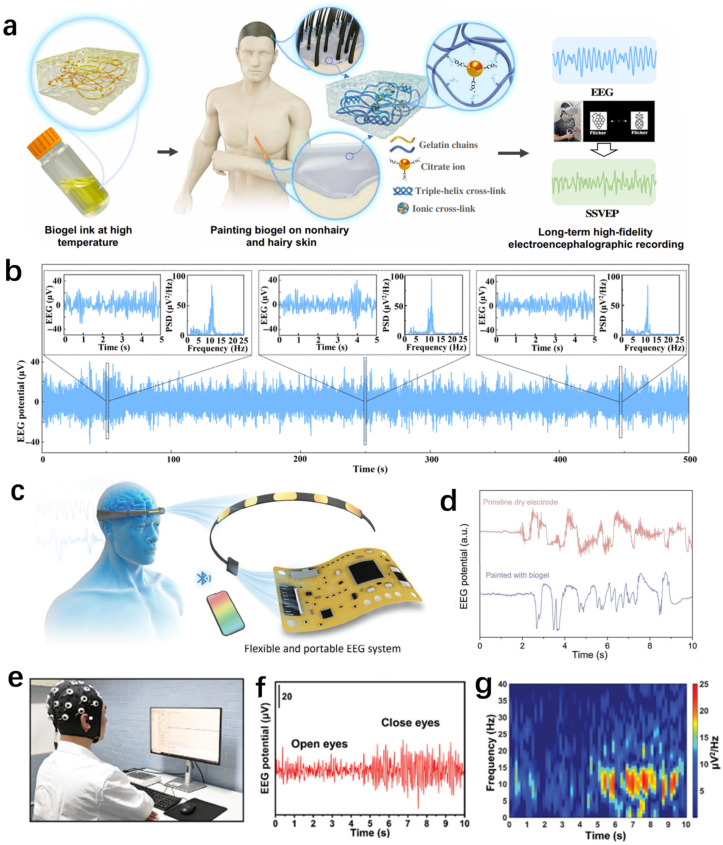
Characterization of the properties of various natural-polymer-based hydrogels and their application in EEG monitoring. (**a**) Schematic representation of the concept of a coatable biogel on a hairy scalp for recording EEG. (**b**) Long-period EEG alpha rhythms continuously recorded by the painted biogel electrode [155]. (**c**) Schematic illustration of the portable EEG acquisition head ring for wireless transmission. (**d**) The EEG artifact signals with blinking collected by electrodes painted with and without biogel [87]. (**e**) The schematic diagram of EEG signal monitoring. (**f**) EEG signals recorded by the MXene/HA-PBA/TA hydrogel electrode in the eyes-open/closed paradigm. (**g**) The corresponding spectrogram showing clear alpha rhythm in the eyes-closed state [101].

EEG is a non-invasive technique used to measure the electrical activity generated by the brain, such as sleep monitoring, epilepsy monitoring, etc. [155,156,157]. Compared with the two electrophysiological signals mentioned above, EEG signals are relatively weak and difficult to record [158] To achieve a correct and long-term recording of the EEG signals, the neuro-electrodes are required to have low impedance and high charge transfer, biocompatibility and Young’s modulus similar to soft tissues [159,160,161,162]. Thus, the development of hydrogels made of natural polymers as coatings of neuro-electrodes for EEG sensors has attracted more and more attention [163,164]. Someya et al. proposed a conductive dermal paintable biogel which can enable conformal contact between the electrode and the hairy scalp, as shown in Figure 9a [155]. It can be used for high-quality long-term EEG monitoring, as shown in Figure 9b. Ying et al. fabricated an all-natural ionic biogel by a simple method [87]. The biogel has high electrical conductivity and flexibility, complete degradability and temperature-controlled phase change properties for long-term, high-fidelity electrophysiological signal recording (Figure 9c,d). Zhang et al. designed a conformal biohydrogel epidermal sensor [101]. This biohydrogel sensor has excellent self-adhesion, high signal-to-noise ratio and low interfacial impedance for high-quality monitoring of EEG signals (Figure 9e). Figure 9f,g show the EEG signals and corresponding time-frequency spectrograms recorded by this biohydrogel epidermal sensor in the eye-open and eye-closed states. A very clear α-rhythm can be found in the eye-closed condition, while no α-rhythm is seen in the eye-open condition. By mixing various polysaccharide materials, Tomić et al. made a conductive nanocomposite hydrogel with good adhesion and self-repairing properties, which was used in electrophysiological monitoring such as EEG monitoring [158]. In summary, natural-polymer-based hydrogels are widely used for EEG signal monitoring due to their excellent electrical conductivity, self-healing properties and adhesion.

## 4. Conclusions and Future Perspectives

Natural polymers have been recognized as ideal materials for preparing hydrogels that are biocompatible, degradable, non-toxic and low-cost, while also solving the environmental issues and waste of resources caused by the use of non-degradable materials. In this report, firstly, we discuss the prospects of natural-polymer-based hydrogels for flexible electronics, and then we describe in detail different natural polymers including proteins and polysaccharides and their methods of preparation. Finally, we provide a detailed description of the application of natural-polymer-based hydrogels for flexible electronics in human movement detection and electrophysiological signal monitoring. Due to the good electrical conductivity and conformability of natural-polymer-based hydrogels, the contact impedance between flexible electrodes and living organisms is minimized, and long-term, stable physiological signals can be monitored.

Although the preparation and application of natural-polymer-based hydrogels have made significant progress, there are still some challenges and issues waiting to be addressed. To achieve specific traits such as excellent mechanical properties, high electrical conductivity and strong adhesion, natural polymer materials are blended with synthetic materials. However, this approach tends to reduce the biodegradability and biocompatibility of natural hydrogels. Therefore, it is crucial to develop hydrogels based on entirely natural polymeric materials, which are capable of exhibiting excellent properties without relying on other synthetic functional materials. Secondly, the preparation methods of natural-polymer-based hydrogels are still in the early stages of research and development, and their relatively low performance makes it difficult for them to compete with commercial products. The development of low-cost and environmentally friendly technologies is urgently needed to continuously improve the performance of natural-polymer-based hydrogels for practical applications. Thirdly, limitations in the performance of natural-polymer-based hydrogels compared to artificial polymer hydrogels restrict the range of further applications, including the development of flexible electronic devices. That is a major issue that needs to be addressed at this point in time. In summary, although many challenges are still faced by fully biophilic natural polymer materials, research and development of natural-polymer-based hydrogels are opening up a new era of flexible electronics. With their specific functionalities being continuously enhanced, these natural hydrogels are expected to play an important role in areas such as flexible e-skin and wearable human–machine interaction (HMI), and lay the foundation for the next generation of high-performance, sustainable and biodegradable hydrogels.

## Data Availability

Data sharing not applicable.

## Abbreviations

**Abbreviation**	**Full Name**
ECG	electrocardiogram
EMG	electromyogram
EEG	electroencephalogram
SEM	scanning electron microscope
SF	silk fibroin
CNFs	cellulose nanofibrils
Gly	glycerol
TA@CNF	tannic acid-decorated callulose nanofibrils
PTCM-Gly	PAAm-TA@CNF-MXene-Gly
TA	tannic acid
PAAm	polyacrylamide
PAAm SN	single network polyacrylamide hydrogel
DN	double network
fc-DN	fiber-connected dual networks
f-DN	fibrillar dual networks
C-DN	connected dual networks
DA	degree of acrylation
AG	agarose
AcAG	acrylated agarose
AAm	acrylamide
HA	hyaluronic acid
Ti_3_C_2_T_x_	MDlene
PBA	phenylboronic acid
TA	tannic acid
PBS	phosphate buffered saline
HMI	human-machine interaction
PCA-Na	sodium pyrrolidone carboxylic acid
